# Anasarca, steatorrhea, and hypoalbuminemia 18 years after total gastrectomy: a case report

**DOI:** 10.1186/s40792-019-0721-7

**Published:** 2019-10-24

**Authors:** Yu Igata, So Okubo, Yu Ohkura, Masaki Ueno, Harushi Udagawa

**Affiliations:** Department of Gastroenterological Surgery, Toranomon Hospital, 2-2-2 Toranomon, Minato-ku, Tokyo 105-8470 Japan

**Keywords:** Total gastrectomy, Anasarca, Hypoalbuminemia, Pancreatic exocrine insufficiency, Capillary leak syndrome, Pancrelipase

## Abstract

**Background:**

Pancreatic exocrine insufficiency (PEI) is known to occur after total gastrectomy. We experienced a case of PEI occurring 18 years after surgery, leading to a potentially fatal condition of capillary leak syndrome (CLS).

**Case presentation:**

The case is a 58-year-old man on a healthy diet who underwent total gastrectomy 18 years before. He was admitted for a 3-month history of anasarca, steatorrhea, and hypoalbuminemia. An episode of fever occurred during workup, followed by pulmonary edema and shock. The patient was transferred to the intensive care unit and was started on fluid management with albumin infusion. A multidisciplinary team meeting was held, and a clinical diagnosis of PEI resulted in CLS was made and we started administration of oral pancrelipase to show clinical improvement. The patient was discharged, and he remained asymptomatic for 13 months.

**Conclusion:**

In a post-gastrectomy patient with malnutrition, PEI should be suspected regardless of the period since surgery. When recognized, immediate replenishment of albumin and pancreatic enzymes should be initiated to prevent clinical deterioration.

## Background

Pancreatic exocrine insufficiency (PEI) is known to occur after total gastrectomy, and it commonly manifests as steatorrhea, malnutrition, and weight loss [1]. However, diagnosis of a patient presenting with anasarca, steatorrhea, and hypoalbuminemia 18 years after total gastrectomy is challenging. We experienced a case of unrecognized PEI which resulted in pulmonary edema and shock, constituting capillary leak syndrome (CLS), a constellation of disease manifestations associated with increased capillary permeability [2]. The patient was successfully treated with fluid management and administration of oral pancrelipase, a porcine pancreatic extract containing lipases, proteases, and amylases.

## Case presentation

**Fig. 1:**
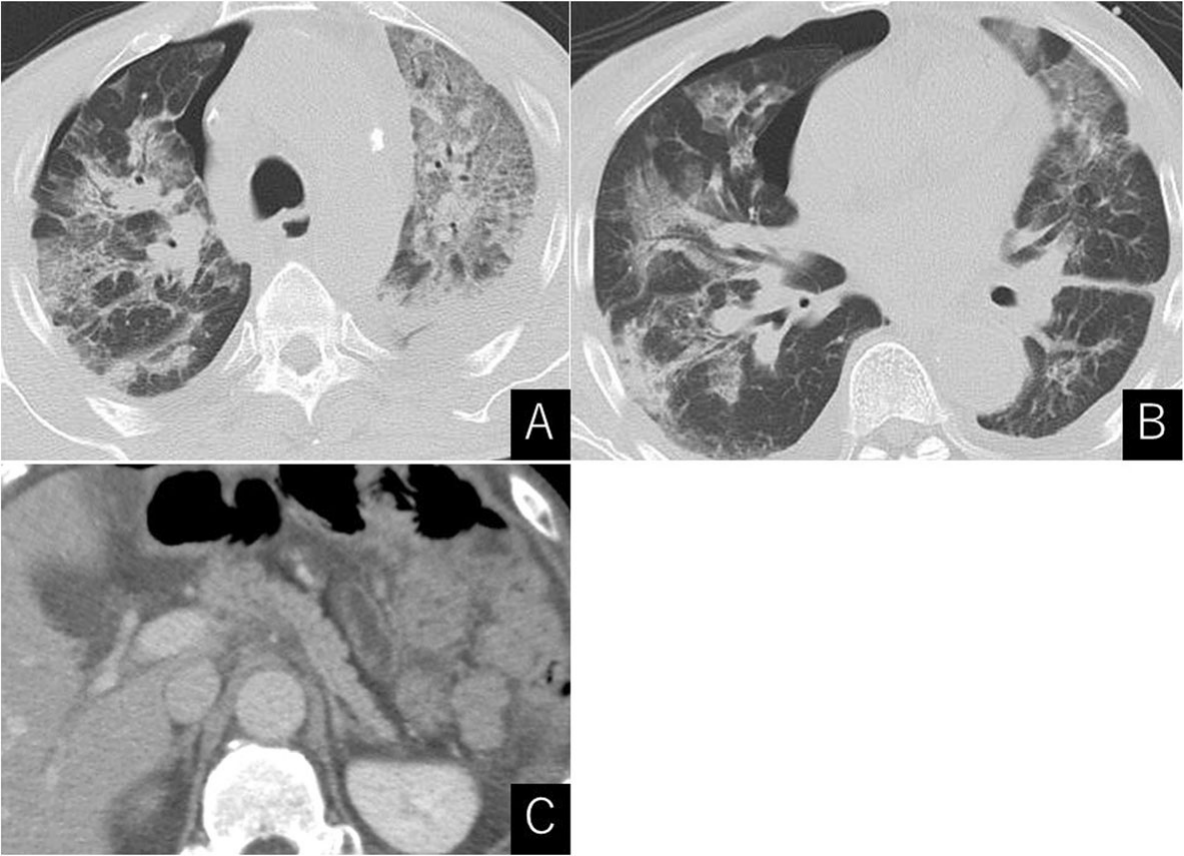
CT scan showed severe bilateral pulmonary edema. Small amount of iatrogenic pneumothorax was secondary to thoracentesis (**a**, **b**). Pancreas was intact (**c**)

**Fig. 2:**
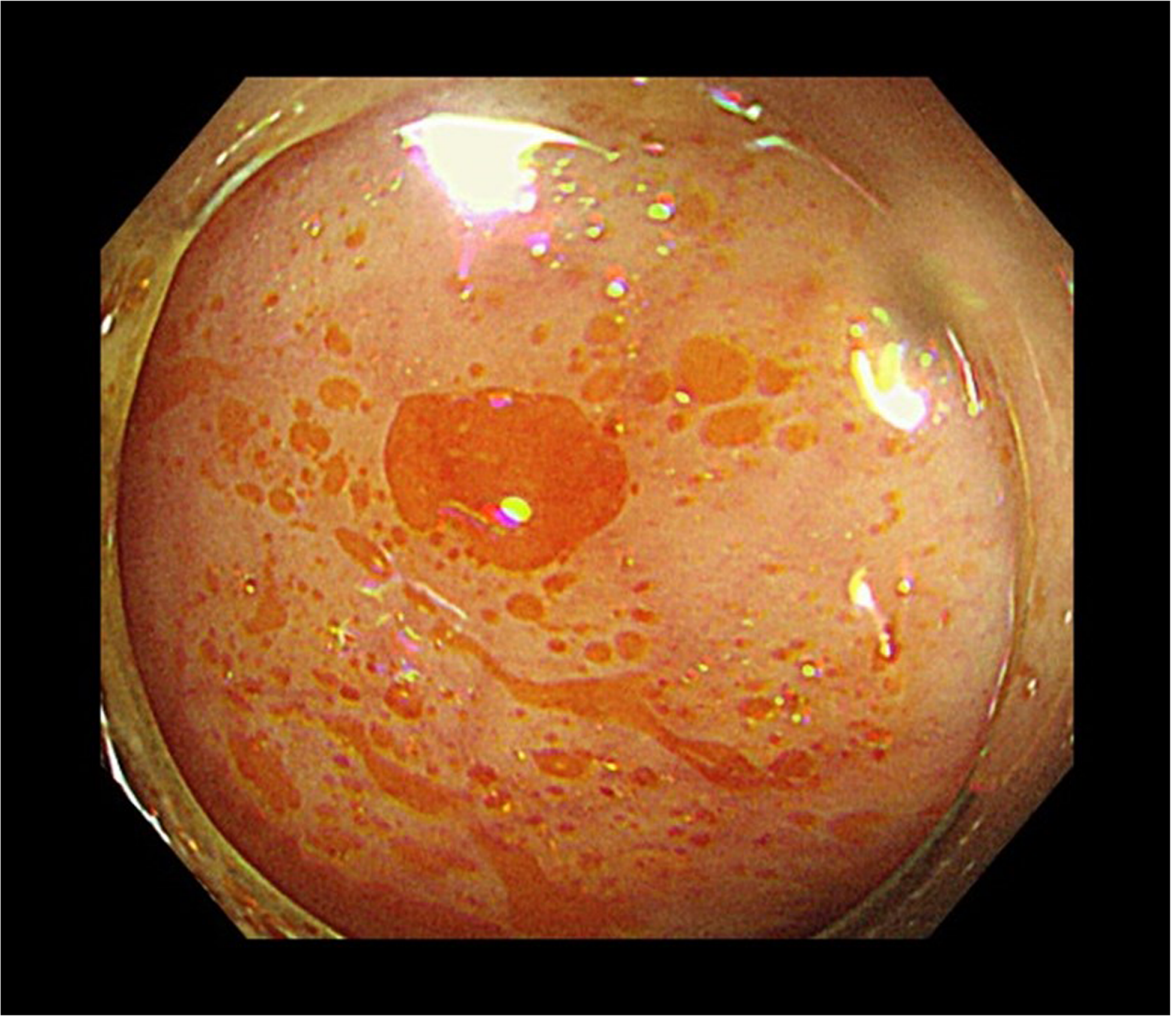
Colonoscopy showed prominent oil droplets

**Fig. 3:**
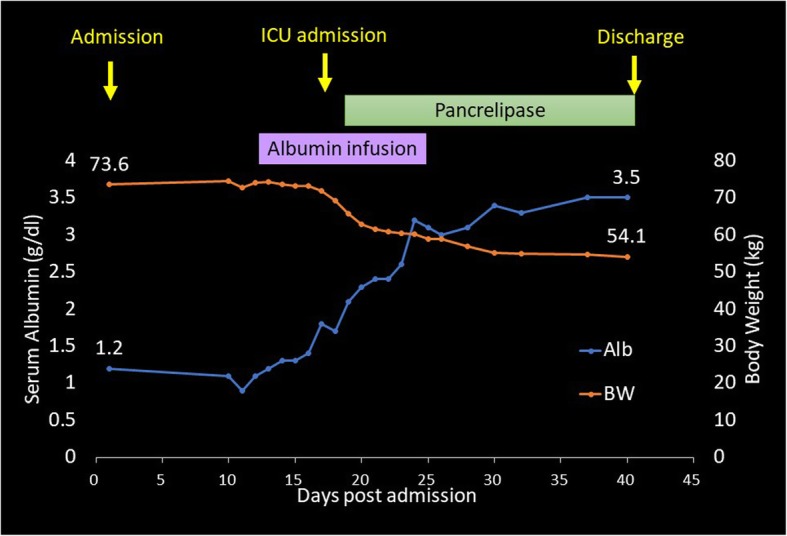
Clinical course with transitional changes in the serum albumin level and body weight

A 58-year-old man with a history of an advanced gastric cancer treated by total gastrectomy and splenectomy and Roux-en-Y reconstruction with D2 lymph node dissection 18 years before was admitted with a 3-month history of anasarca, steatorrhea, and hypoalbuminemia. He had been taking vitamin supplementations and had been followed up annually for his gastric cancer with no signs of recurrence. He had a medical history of stroke 3 years before. As for social history, he drank up to 300 g of alcohol a day until 42 years old, and thereafter, he cut down to 80 g of alcohol a week. He was on a regular diet, and he was never diabetic. On presentation, his serum albumin was 1.2 g/dl (reference range 4.1–5.1 g/dl), vitamin B_12_ was 1410 ng/L (233–914 ng/L), and folic acid was 5.8 μg/L (3.6–12.9 μg/L) (Table 1). Computed tomography showed pleural effusions and ascites. Thorough examinations were scheduled, where he developed fever, bilateral pulmonary edema, and shock (Fig. 1). The patient was transferred to the intensive care unit and was started on immediate albumin resuscitation. As the blood cultures were negative, this “sepsis-like” syndrome with hypoalbuminemia, anasarca, pulmonary edema, and shock was consistent with the clinical diagnosis of CLS. Due to the rapid clinical deterioration from an undiagnosed etiology, a multidisciplinary team meeting was held. From the results of the multiple examinations (Table 2), a diagnosis of PEI was suspected, and an oral daily dose of 1800 mg of pancrelipase was initiated (Fig. 2). The patient gradually improved and the clinical signs of CLS resolved (Fig. 3). The patient was discharged and is routinely followed up, remaining asymptomatic for 13 months.

**Table 1:** Laboratory data on admission

Laboratory data					
WBC	9200	/μl	Na	138	mEq/l
RBC	249 × 10^4^	/μl	K	4.0	mEq/l
Hgb	8.9	g/dl	Cl	108	mEq/l
Hct	25.9	%	Ca	7.3	mg/dl
PLT	37.4 × 10^4^	/μl	Glucose	78	mg/dl
			HbA1c	3.4	%
PT%	58.0	%			
APTT	34.2	Sec	CEA	17.0	μg/l
			CA19-9	47	U/ml
TP	4.5	g/dl	DUPAN-2	276	U/ml
Alb	1.2	g/dl	Span-1	45.0	U/ml
AST	33	IU/l	sIL-2R	563	U/ml
ALT	36	IU/l			
LD	329	IU/l	Vitamin B_12_	1410	ng/l
γ-GTP	18	IU/l	Folic acid	5.8	μg/l
ALP	234	IU/l	Zn	27	μg/dl
AMY	30	IU/l			
CK	147	IU/l			
BUN	20	mg/dl			
Cre	0.87	mg/dl			
T-bil	0.7	mg/dl			
CRP	2.0	mg/dl

**Table 2:** Results of core examinations performed

Performed examinations	Brief findings
Contrast enhanced CT	Fatty liver. No tumors detected
MRI	No tumors detected
Endoscopic ultrasonography	No tumors detected
Colonoscopy	Floating oil droplets were evident (Fig. 2)
Small intestine endoscopy	No evidence of Crohn’s lymphangiectasia
Albumin scintigraphy	No evidence of albumin loss
Rectal biopsy	No amyloid deposits
Bone marrow biopsy	No evidence of lymphoma

*CT* computed tomography, *MRI* magnetic resonance imaging

## Discussion

Pancreatic exocrine insufficiency presenting with anasarca 18 years after total gastrectomy, associated with a potentially fatal course of CLS, has not been reported in the previous literatures.

Although PEI after total gastrectomy is a well-documented complication [1, 3–5], there is a paucity of publication focused on the timing of the onset of malnutrition-associated symptoms after gastrectomy. Therefore, suspecting a relatively late onset of PEI in this rare clinical setting was a challenge, which leads to a diagnostic delay. From the examination results, the malnutrition state could not be attributed to malignancy, infection, autoimmune diseases, amyloidosis, nephrotic syndrome, liver failure, Crohn’s disease, Celiac disease, and other causes of hypoalbuminemia, which lead to a clinical diagnosis of PEI. Furthermore, the fact that the patient is remaining asymptomatic on pancrelipase supports PEI as the underlying etiology.

This is also the first report on untreated PEI resulting in bilateral pulmonary edema and shock leading to the diagnosis of CLS. Capillary leak syndrome has been used to describe this rare constellation of disease manifestations associated with increased vascular permeability to proteins, i.e., edema, cavity effusions, non-cardiac pulmonary edema, and hypotension. It is divided into two categories, idiopathic or secondary, based on the underlying diseases [2]. The relationship between PEI and CLS is unclear, but the condition of PEI could have played a role in developing CLS. Therefore, we hypothesized that by recognizing PEI and initiating treatment with pancrelipase and albumin infusion as needed, progression to CLS could be prevented.

As alteration of normal anatomy and pancreatic denervation is reported to be responsible for PEI, patients operated on the esophagus, duodenum, and pancreas are also at risk for developing maldigestion [3, 4]. In patients with malnutrition-associated symptoms with histories of upper gastrointestinal surgery, PEI should be sought regardless of time from surgery. Although previous reports on the efficacy of pancreatic enzyme replacement therapy have been controversial [5], given the profound severe consequences, administering pancrelipase in this patient population is thought to be acceptable.

## Conclusions

Pancreatic exocrine insufficiency can occur 18 years after total gastrectomy and can present with anasarca. Untreated PEI harbors risks of developing a potentially fatal clinical condition of CLS. Surgeons must suspect PEI and initiate albumin and pancrelipase replenishment as needed without hesitation.

## Data Availability

The dataset supporting the conclusions of this article is included within the article.

## Abbreviations

CLS: Capillary leak syndrome
CT: Computed tomography
MRI: Magnetic resonance imaging
PEI: Pancreatic exocrine insufficiency
